# Process and Properties of Al-Mg-Er-Zr-Sc High-Strength Aluminum Alloy Powder Prepared by Vacuum Induction Melting Gas Atomization

**DOI:** 10.3390/ma18081763

**Published:** 2025-04-11

**Authors:** Zhengjiang Gao, Fei Zhang, Hui Li, Teng Ma, Huan Yang, Wei Wang, Wu Wei, Shengping Wen, Hui Huang, Xiaolan Wu, Kunyuan Gao, Li Rong, Xiangyuan Xiong, Zuoren Nie

**Affiliations:** 1 State Key Laboratory of Materials Low-Carbon Recycling, Beijing University of Technology, Beijing 100124, China; 2 Avimetal AM Tech Co., Ltd., Beijing 100176, China; 3 Avimetal AM Tech Co., Ltd., Xuzhou 221221, China

**Keywords:** AlMgErZrSc high-strength alloy, VIGA, gas atomization, numerical simulation, additive manufacturing

## Abstract

The Er-Zr-Sc-modified Al-Mg alloys produced by additive manufacturing (AM) exhibit good formability and excellent mechanical properties, and present great potential for applications in the fields of aerospace and automotive fields. In this work, the preparation process of Al-4.5Mg-0.7Er-0.5Zr-0.3Sc high-strength aluminum alloy powder for additive manufacturing by vacuum induction melting gas atomization (VIGA) was investigated. With the goal of obtaining excellent sphericity and higher powder yield in the particle size range of 15~53 μm, a new type atomizer with optimized convergence angle and tube extension length was designed based on finite element numerical simulation and experimental research, and the optimal atomization processing parameters were determined. The results revealed that when the convergence angle was 32° and the extension length was 5 mm, the large negative pressure and suction force at the tube outlet could facilitate the smooth flow of the melt and a refined powder particle size; when the melt temperature was 800 °C and the atomization pressure was 3.25 Mpa, the melt had low viscosity and the atomization gas could fully interact with the melt. Meanwhile, the melt droplets had suitable cooling conditions, avoiding the generation of irregular powders and improving the powder sphericity. Under the above optimal processing parameters, the prepared powders were spherical or nearly spherical with fine particle size and a high yield of about 39.45%.

## 1. Introduction

Due to their high specific strength, excellent corrosion resistance, and good ductility, Al-Mg alloys are widely used in the aircraft, shipbuilding, and automotive fields. With the development of aerospace and new energy vehicles, components need to meet higher requirements for strength and serviceability, and at the same time, the structure of components is becoming increasingly complex. As a typical additive manufacturing technology, laser powder bed fusion (LPBF) technology based on the principle of layer-by-layer manufacturing has been rapidly promoted and applied in recent years due to its ability to achieve rapid and integrated manufacturing of complex components [1]. Thus, the development of new high-strength aluminum alloys that are compatible with LPBF has become a global research hot spot.

By adding Sc and Zr elements to Al-Mg alloys, Airbus has developed Al-Mg-Sc-Zr high-strength aluminum alloys that have good compatibility for additive manufacturing and exhibit excellent performance with a strength of over 520 MPa, an elongation of over 8.6%, and almost no anisotropic effect after heat treatment [2]. The relevant enterprises or institutes in Table 1 have successively launched high-strength aluminum alloy materials such as Al-Mg- and Al-Cu-based alloys and conducted extensive research on their chemical composition design, process parameters, heat treatments, microstructure, and mechanical properties [2,3,4,5,6]. However, due to the high price of Sc, AlMgScZr high-strength aluminum alloy powder is expensive, which to some extent limits its commercial application in industrial fields.

The vacuum induction melting gas atomization (VIGA) method is one of the main processes for the preparation of spherical aluminum alloy powders with low oxygen and high purity [7]. Our team prepared a new type of Er-Zr-Sc composite strengthened Al-Mg alloy powder using the VIGA process in the early stage, which was suitable for additive manufacturing and could be used to fabricate crack-free samples by the LPBF process. Studies have been conducted on the microstructure and mechanical properties of this LPBF-processed alloy, indicating that Er has a similar effect with Sc and Zr elements in aluminum alloys. Al_3_M (M is one or several elements of Er, Sc, and Zr) particles formed during the LPBF process could act as heterogeneous nucleation sites to promote the formation of equiaxed grains, which can improve the LPBF formability, and the nano-sized Al_3_M particles generated during aging can greatly improve the mechanical properties. Replacing some of the Sc elements with inexpensive Er elements not only reduces raw material costs but also ensures the high strength of the alloy [8,9,10].

Although the addition of rare elements such as Er, Zr, and Sc improves the formability and mechanical properties of aluminum alloys, it brings great difficulties to the preparation of aluminum alloy powders by VIGA technology. On the one hand, because Er, Zr, and Sc elements are easy to oxidize, a higher purity atmosphere is required in the preparation process. On the other hand, these elements have significantly higher melting points than Al, which increases the viscosity of the aluminum alloy melt. This is not only detrimental to the control of the sphericity of powders but also affects the smooth pouring of melt as well as the atomization process, which ultimately affects the particle size and powder yield within the size range of 15~53 μm (the most suitable particle size range for additive manufacturing). The yield of the aluminum powder prepared by our team using the existing atomizer nozzle and process parameter in the early stage was about 28%. Considering the huge demand for lightweight and high-strength alloys in industries such as aerospace and new energy vehicles, Al-Mg-Er-Zr-Sc high-strength aluminum alloy powder for additive manufacturing has great market and application prospects. Therefore, it is necessary to conduct in-depth research on the fabrication process by VIGA to improve the powder particle size distribution as well as its sphericity. In this research, the atomizer, which was the key component in the powder preparation process, was redesigned. The effects of the convergence angle of the atomizer nozzle and extension length of the melt delivery tube on powder properties were studied through numerical simulation and experimental methods, and a new type of atomizer suitable for Al-Mg-Er-Zr-Sc alloy was developed. Moreover, the effects of melt temperature and atomization pressure on powder particle size, powder yield, and morphology were analyzed, and the optimal preparation process parameters for Al-Mg-Er-Zr-Sc alloy by VIGA were determined.

## 2. Numerical Models and Calculation Methods

As a widely used powder production technology, VIGA can prepare aluminum alloy powders with low oxygen content and high sphericity [11,12]. However, the complexity of the VIGA process cannot be ignored. The atomization of the metal melt is a complex physical process with multi-parameter coupling [13,14]. Many scholars have studied the atomization process from different perspectives. The Lubanska formula [15] is currently widely recognized as an empirical formula for calculating atomized particle size:

(1) $$d_{50}=kd_{0}{\left[\left(1+\frac{M}{G}\right)\frac{V_{l}}{V_{g}We}\right]}^{1/2}$$

(2) $$W_{e}=\frac{\rho_{l}{∆U}^{2}D}{\sigma }$$

In Equation (1), $d_{50}$ is the median particle size, which is an important indicator reflecting the overall particle size of the powder, k is the empirical constant related to the diameter of the delivery tube, $d_{0}$ is the diameter of liquid metal flow, a is the empirical constant related to the injection angle, M/G is the mass flow ratio of liquid metal to atomization gas flow, $V_{l}$ and $V_{g}$ are the kinematic viscosity coefficients of metal liquid and gas, respectively, and $W_{e}$ is the Weber number. In Equation (2), $\rho_{l}$ is the density of the metal liquid, U is the relative velocity between the atomization gas and liquid metal, σ is the surface tension of the liquid, and D represents the diameter of the molten liquid column, which is usually replaced by the inner diameter of the melt delivery tube. Based on the above equation, it can be concluded that for a specific material, the powder particle size is closely related to the diameter of liquid metal flow, the gas flow velocity, and the mass flow ratio of liquid metal to gas flow. Increasing the atomization gas velocity can reduce the mass flow ratio of liquid metal to gas flow, thereby reducing the value of $d_{50}$. Therefore, finer powder particles can be obtained by increasing the velocity of atomization gas and reducing the viscosity of metal liquid [16].

**Figure 1:**
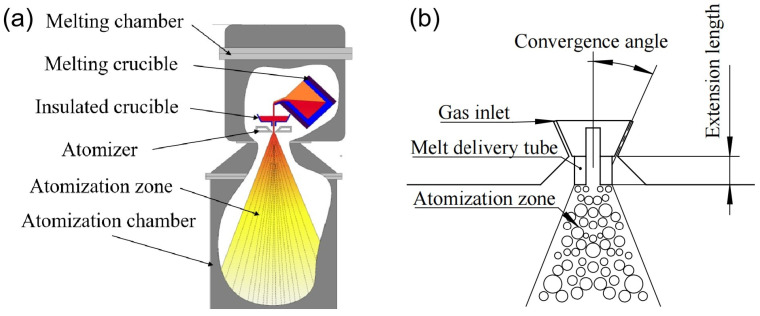
Schematic diagram of powder preparation by VIGA (**a**) and the atomizer structure (**b**).

The velocity of atomization gas is closely related to the convergence angle of the atomizer nozzle and the extension length of the melt delivery tube [17,18,19]. The principle of powder preparation by VIGA is shown in Figure 1. In order to obtain preliminary parameters of convergence angle and extension length, firstly, based on the Computational Fluid Dynamics (CFD) method, the influence of convergence angle on the characteristics of the gas flow field was simulated under a fixed extension length, and a suitable nozzle convergence angle was obtained. Then, the gas flow field with different extension lengths under the optimized convergence angle was simulated, and the extension length that can lead to higher gas velocity and larger negative pressure at the delivery tube outlet was selected. In order to verify the simulation results and further analyze the effects of convergence angle and extension length on the powder size and yield, powder preparation tests were conducted at different convergence angles and extension lengths, and the optimal atomization process parameters were determined.

**Figure 2:**
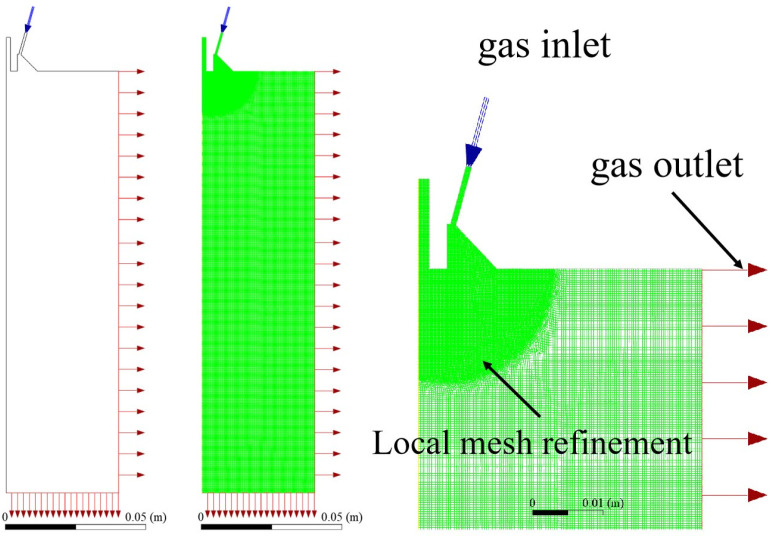
Computational domain and mesh division.

In order to obtain the optimal convergence angle and extension length parameters, the characteristics of the gas flow field under different convergence angles and extension lengths were studied using the CFD method. A two-dimensional axisymmetric model was adopted to study the gas flow field under different parameters. Inlet and outlet boundary conditions of pressure were set [17], and the mesh near the gas outlet was encrypted. By using steady-state calculation in Fluent software (Fluent 2021R1), argon gas was set as the ideal gas, and the k—ω SST model was adopted. The central axis of the model was set as the symmetrical boundary, the gas inlet was set as the pressure inlet, the bottom and side edges of the model were set as the pressure outlet, and the remaining boundaries were assumed to be the wall surface. A pressure-based solver was chosen and iterative calculations were performed using the SIMPLE algorithm. The computational domain and mesh division are shown in Figure 2.

## 3. Experimental Design and Methods

### 3.1. Powder Preparation

The VIGA100 equipment, manufactured by Avimetal AM Tech Co., Ltd. (Beijing, China), was used to fabricate aluminum alloy powder. The designed chemical composition is shown in Table 2. Pure aluminum and binary alloys such as AlMg15, AlEr20, AlZr10, AlSc2, AlFe20, AlSi20, AlMn20, etc., were adopted as raw materials.

The raw materials were melted using an intermediate frequency induction power supply in a high-purity argon protection atmosphere, and the melt temperature was measured using a thermocouple. When the melt temperature reached the set temperature (750~850 °C), the melt in the crucible was poured into the intermediate package, and then the gas atomization process began. The atomization gas was 99.999% high-purity argon with an atomization pressure of 3.0~3.5 MPa.

### 3.2. Powder Performance Testing

The prepared Al-Mg-Er-Zr-Sc aluminum alloy powder was sieved, and then tested for chemical composition, particle size, powder morphology, apparent density, and tap density.

An inert gas-protected vibrating screen and airflow classifier were used to sieve the prepared powders, with the vibrating screen removing particles larger than 53 μm and the airflow classifier removing particles smaller than 15 μm. Then, the powder with a particle size distribution of 15~53 μm was obtained, and the ratio of the obtained powder mass to the total powder mass was the powder yield.

A laser particle size analyzer (Mastersizer 3000, Malvern Panalytical, London, UK) was used for particle size analysis. There are three key indicators for evaluating particle size, namely D10, D50, and D90, which refer to the values of powder particle size corresponding to the cumulative proportion of particle size in the powder sample reaching 10%, 50%, and 90%, respectively. D50, which is also known as median particle size, has a physical meaning that the proportion of powder particles with a particle size smaller than the value of D50 is 50%. D50 is an important indicator to reflect the overall particle size of the powder, and D10 and D90 reflect the proportion of small-size and large-size powders, respectively.

The chemical composition of the powder was examined using inductively coupled plasma atomic emission spectroscopy (ICP-AES, Plasma 3000, NCS, Beijing, China) and an oxygen–nitrogen analyzer (ON 3000, NCS, Beijing, China). Powder density was tested using an apparent density meter (BT101, Bettersize, Dandong, China) and a tap density meter (BT311, Bettersize, Dandong, China). A scanning electron microscope (SEM, JSM-7900F, JEOL, Tokyo, Japan) equipped with energy-dispersive X-ray spectroscopy (EDX) was utilized to observe the powder morphology and sphericity, and map scanning analysis was conducted to analyze the chemical compositional segregation of the powder particles.

### 3.3. LPBF Process

The powder with particle sizes ranging between 15 and 53 μm was used for LPBF forming, and it was processed on a commercial MT280 powder-bed machine (Avimetal AM Tech Co., Ltd. (China)). Before the LPBF processing, an Al substrate was fixed on the platform and preheated to 100 °C. The Al-Mg-Er-Zr-Sc alloy powder with a thickness of 30 μm was deposited on the substrate by powder coating scraper. Then, the laser beam began to scan the powder bed according to the CAD model. The steps of powder laying and laser scanning were performed repeatedly after the first layer scanning was finished. The whole process was carried out in an Ar protective atmosphere. The processing parameters are displayed in Table 3. The formed specimens were horizontal and vertical bars with a dimension of Φ13 mm × 75 mm, which were subjected to aging treatment at 350 °C for 2 h in a muffle furnace, and then tested on a universal tensile testing machine (E45.105, MTS, Shanghai, China) with a strain rate of 2.3 × 10^−3^/s at room temperature.

## 4. Experimental Results and Discussion

### 4.1. Numerical Calculation Result and Experimental Verification

#### 4.1.1. Research on Convergence Angle of Atomizer Nozzle

The influence of the nozzle convergence angle on the single-phase gas flow field was simulated and studied. The convergence angles ranged from 28° to 48°. The external diameter and the extension length of the melt delivery tube were 12 mm and 6 mm, respectively. The atomization pressure was set to 3 MPa. The specific parameters are displayed in Table 4.

**Figure 3:**
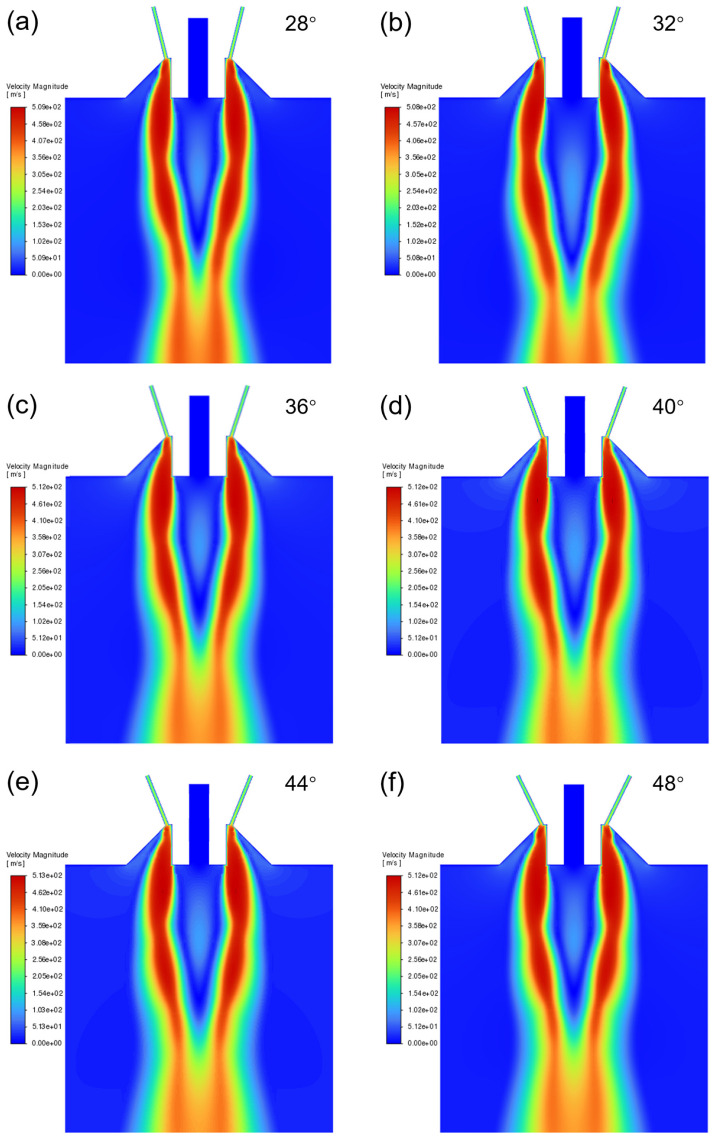
Gas flow velocity field under different convergence angles: (**a**) 28°; (**b**) 32°; (**c**) 36°; (**d**) 40°; (**e**) 44°; and (**f**) 48°.

The gas flow velocity field under different nozzle convergence angles is shown in Figure 3. It can be seen that the gas flow field with different convergence angles presents similar structural characteristics. The atomization gas expands to form a supersonic airflow when passing through the outlet of the atomizer nozzle, leading to the formation of an inverted conical recirculation zone below the outlet of the melt delivery tube. The high-speed gas flow has a strong impact on the metal melt, breaking up the melt into small droplets [20,21,22]. There are some differences in the shape of the recirculation zone formed under different convergence angles, which affects the negative pressure at the outlet of the melt delivery tube and also its suction effect on the metal liquid.

**Figure 4:**
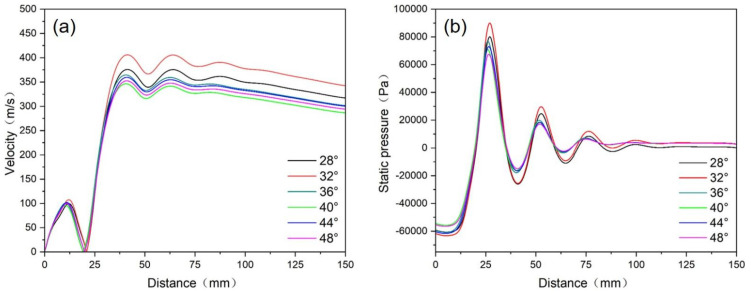
The velocity distribution (**a**) and pressure distribution (**b**) of the gas flow along the axis direction under different convergence angles.

Figure 4 shows the velocity distribution and pressure distribution of the gas flow along the axis direction, respectively. The center point of the outlet of the delivery tube is located at 0 mm on the *x*-axis in the figure. It can be seen that the static pressure in the recirculation zone shows a similar trend in the axial direction under different convergence angles, which gradually increases from the outlet and reaches its maximum at the convergence point and then gradually decreases. Therefore, in order to facilitate the smooth flow of the melt and enhance the action of gas on the melt, it is more appropriate to control the convergence angle at around 28~32°.

#### 4.1.2. Research on the Extension Length of the Melt Delivery Tube

**Figure 5:**
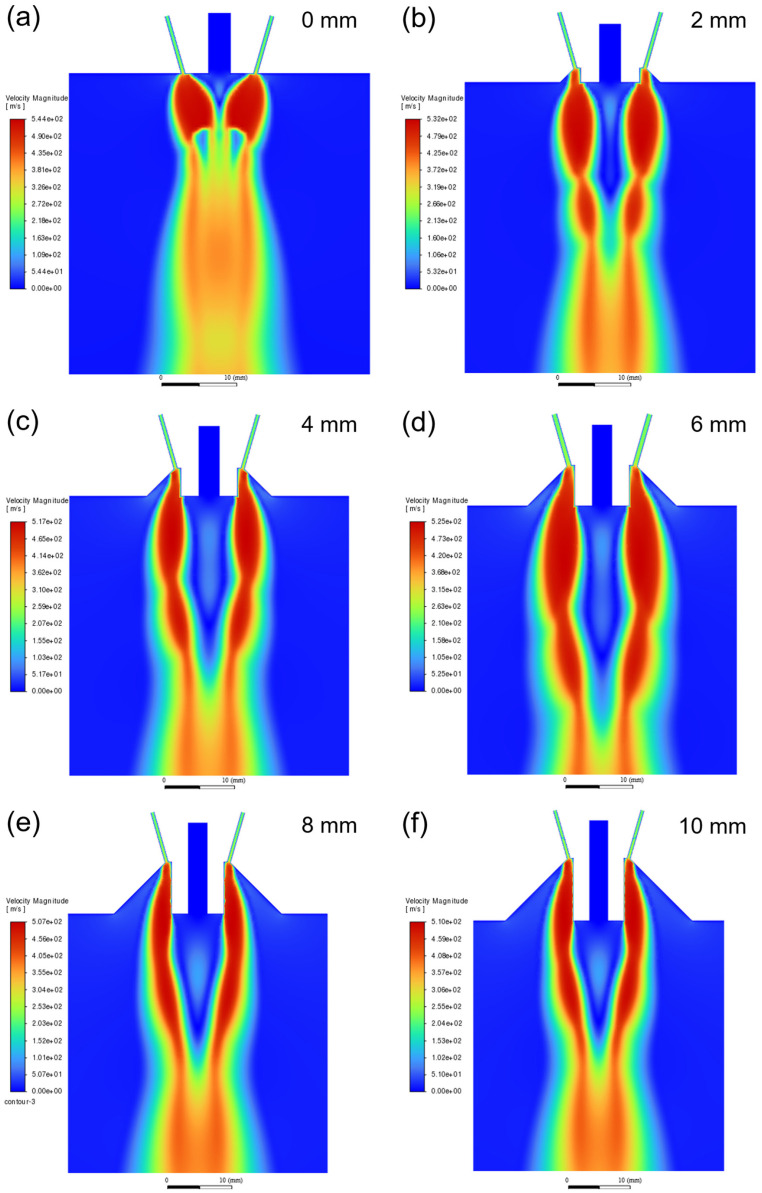
Gas flow velocity field under different extension lengths: (**a**) 0 mm; (**b**) 2 mm; (**c**) 4 mm; (**d**) 6 mm; (**e**) 8 mm; and (**f**) 10 mm.

In order to investigate the effect of the extension length of the melt delivery tube on the gas flow field, the characteristics of the gas flow field under different extension lengths (from 0 to 10 mm) at a constant convergence angle of 32° and atomization pressure of 3 MPa were studied. The gas flow velocity fields under different extension lengths are shown in Figure 5. It can be seen that the longer the melt delivery tube extends, the greater the impact on the gas expansion at the tube outlet. When the extension length is 0 mm, the high-pressure gas expands freely at the tube outlet. When the delivery tube extends, the expansion gas collides with the sidewall of the tube and forms a compression wave, which is reflected towards the free boundary on the other side. The longer the extension length of the tube, the more obvious this situation becomes [23]. The gas expansion zone at the outlet is shaped like a mango when the extension length is 0 mm, while as the extension length increases to 4 mm, the expansion zone becomes gourd-shaped. It is worth noting that when the extension length is over 4 mm, the variation trend of the highest gas velocity in the gas flow field is not significant with the increase in the extension length. Therefore, the change in the extension length does not affect the maximum velocity of the gas.

**Figure 6:**
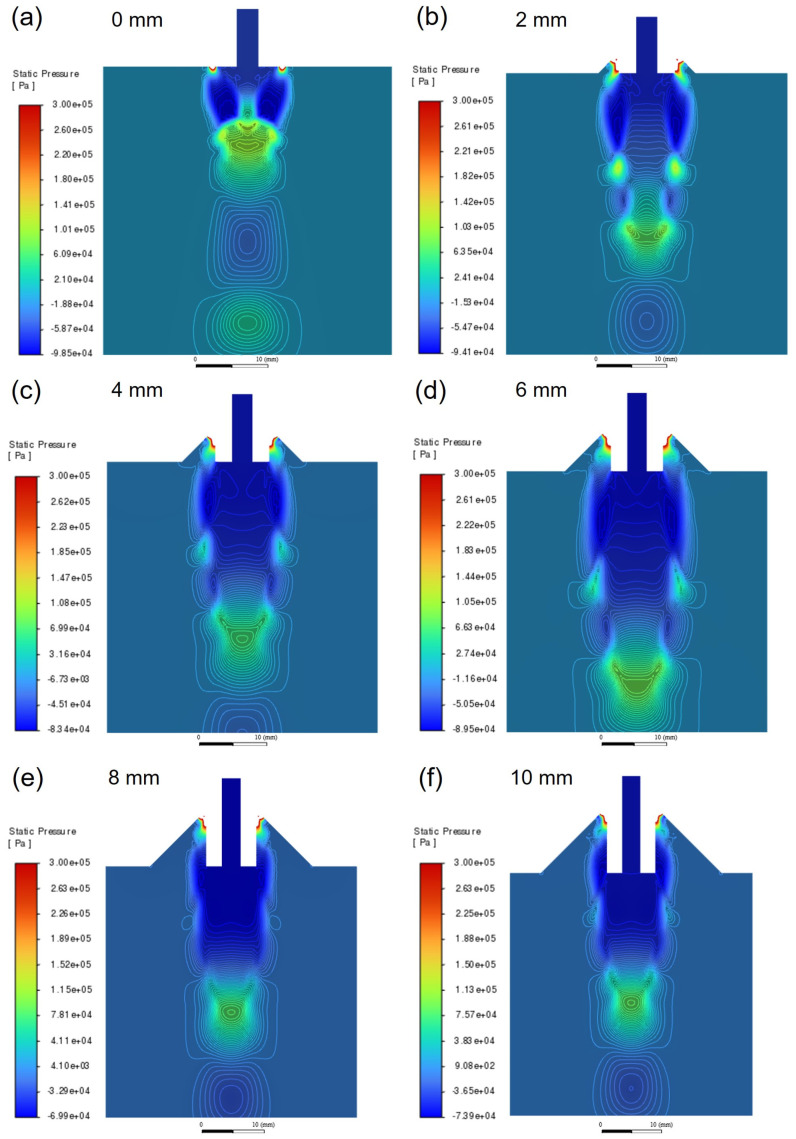
Pressure distribution under different extension lengths: (**a**) 0 mm; (**b**) 2 mm; (**c**) 4 mm; (**d**) 6 mm; (**e**) 8 mm; and (**f**) 10 mm.

Figure 6 shows the pressure distribution near the melt delivery tube outlet under different extension lengths. It can be seen that there is a positive pressure area below the tube outlet. When the extension length is 2~6 mm, the positive pressure area gradually moves downwards. Especially, when the extension length is 6 mm, the distance between the positive pressure area and the melt delivery tube outlet reaches its maximum value, which is about 40 mm. As the extension length increases to 8~10 mm, the positive pressure area gradually shifts upward.

**Figure 7:**
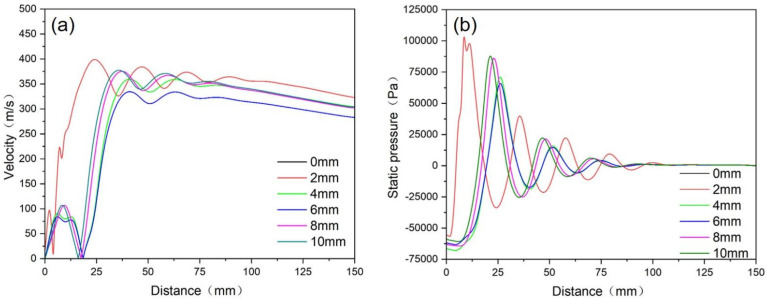
The velocity distribution (**a**) and pressure distribution (**b**) of the gas flow along the axis direction under different extension lengths.

Figure 7 shows the velocity distribution and pressure distribution of gas flow along the axis direction under different extension lengths. In Figure 7a, it can be seen that the center point of the outlet of the melt delivery tube is located at 0 mm on the *x*-axis in the figure. It can be seen that when the tube extension length is 0 mm, there is no point with a gas flow velocity of 0 m/s in the axial direction. The reason is that part of the gas moves along the end of the melt delivery tube head towards the center, changes its motion direction, and moves downwards after reaching the center. When the extension length is 2~10 mm, there is a convergence point of gas flow 10~20 mm downwards from the melt delivery tube outlet, and the gas flow velocity at the convergence point is 0 m/s.

Figure 7b shows a comparison of the absolute pressure curves of gas flow along the axis direction under different extension lengths. Due to the presence of compression waves arising from the expanding gas, the pressure along the axis direction exhibits significant fluctuations. The pressure differential in the axial direction reaches its maximum value when the extension length is 0 mm and reaches its minimum value when the extension length is 6 mm, indicating that the pressure fluctuation along the axis direction will decrease with the increasing extension length.

In order to facilitate the smooth flow of the melt, it is necessary to ensure that there is a negative pressure at the melt delivery tube outlet so as to increase the suction force of gas flow on the melt. From the perspective of improving the atomization efficiency, higher gas flow velocity enables sufficient interaction between gas and metal liquid. Therefore, the extension length of the melt delivery tube should be controlled within an appropriate range, and the ideal extension length should be around 6 mm.

#### 4.1.3. Experimental Verification

In order to verify the simulation results and further investigate the effects of convergence angle and extension length on the powder particle size and powder yield, orthogonal experiments were conducted under a convergence angle range of 28~32° and an extension length range of 5~7 mm, as shown in Table 5.

**Figure 8:**
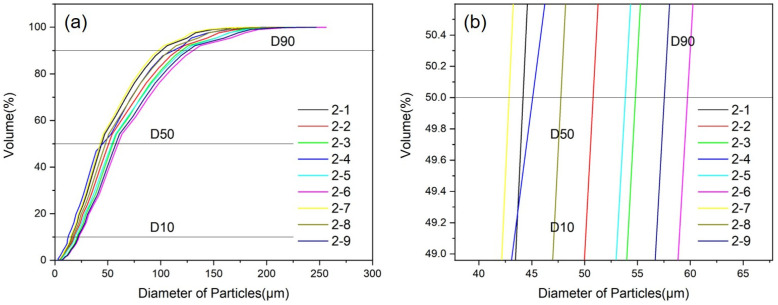
Powder particle size distribution under different convergence angles and extension lengths (**a**) and the enlarged figure (**b**).

The particle size distribution under different convergence angles and extension lengths is shown in Figure 8, and the median particle size and powder yield are shown in Table 6. In Figure 8, it can be seen that the convergence angle and extension length have an impact on the powder particle size distribution. The value of D10 was relatively concentrated, ranging from 10 to 25 μm, while D50 and D90 were relatively broad, with D50 distributed in the range of 44~56 μm and D90 distributed in the range of 95~120 μm. Under the given convergence angle, both the median particle size and the powder yield show an increasing trend with the increase in the extension length of the melt delivery tube. While under the given extension length, the median particle size D50 first decreases and then increases with the convergence angle rising from 28° to 32°, reaching its lowest value at 32°. Based on the gas flow velocity and pressure distribution under different convergence angles and extension lengths, it can be concluded that a short extension length can shorten the distance between the gas jet and metal liquid, which is beneficial for the full interaction between gas and metal liquid. When the extension length is consistent, there is a certain change in the gas flow velocity at the melt delivery tube outlet as the convergence angle increases, which in turn affects the droplet fragmentation effect and then the powder particle size distribution. Under the convergence angle of 32° and extension length of 5 mm (corresponding to 2–7 in Table 6), the powder has the smallest particle size and the highest yield.

### 4.2. Research on Melt Temperature and Atomization Pressure

The melt temperature and atomization pressure are the key factors affecting the powder quality and particle size distribution [24]. The melt temperature has an influence on the viscosity and surface tension of the melt, while the atomization pressure will affect the gas flow velocity and suction pressure [25,26,27]. Therefore, it is necessary to study the particle size distribution and powder yield under different melt temperatures and atomization pressures.

Based on the above simulation and experimental result, the optimum convergence angle and extension length are determined, with a convergence angle of 32° and an extension length of 5 mm. Further experiments are conducted to optimize the melt temperature and atomization pressure. Due to the fact that increasing the melt temperature and atomization pressure can obtain finer powder, the melt temperature is set to 750~850 °C, and the atomization pressure is set to 3.0~3.5 Mpa, and the designed parameters are shown in Table 7.

**Figure 9:**
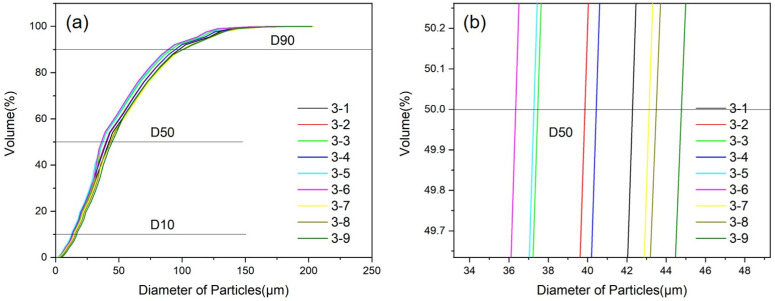
Powder particle size distribution under different melt temperatures and atomization pressures (**a**) and the enlarged figure (**b**).

The prepared powder is sieved out and then tested for particle size and powder yield. Figure 9 displays the particle size distribution of powder, and Table 8 presents the median particle size and the powder yield. In Figure 9, it can be seen that after adjusting the melt temperature and atomization pressure, the powder particle size generally decreases and the size distribution becomes more concentrated, with D10 distributed between 9 and 20 μm, D50 distributed between 35 and 46 μm, and D90 distributed between 75 and 105 μm.

Under a constant melt temperature, the median particle size shows a decreasing trend as the atomization pressure increases, while the powder yield shows an increasing trend. When the atomization pressure is increased from 3.0 MPa to 3.25 MPa, the changes in median particle size and powder yield are significant. However, as the atomization pressure continues to increase, the changes in median particle size and powder yield are relatively small. It is believed that increasing the atomization pressure can increase the gas flow velocity to a certain extent [28]. After reaching a certain point, the gas flow velocity no longer increases with the rising atomization pressure, and simultaneously there exists intense turbulence in the gas flow field, which will affect the flight and solidification process of droplets. The solidified small-sized droplets adhere to the surface of incompletely solidified large-sized droplets to form "satellite" powders [29,30,31], which in turn affects the particle size test results.

As the melt temperature increases from 750 °C to 800 °C, the median particle size decreases, and when it is further increased to 850°, the median particle size increases instead. The higher melt temperature will deteriorate the cooling conditions of atomized droplets and also lead to an increase in “satellite” powder, which is the reason why the particle size first decreases and then increases with the rising temperature. The powder with the smallest median particle size and the highest yield can be obtained at the melt temperature of 800 °C and the atomization pressure of 3.25 MPa. The yield of powder with a particle size of 15~53 μm is up to 39.45%, which is 40.89% higher than before. Based on the above result, it can be concluded that the powder yield can be improved by increasing the melt temperature or atomization pressure in a certain range.

### 4.3. Powder Performance

Based on the above analysis, powder with finer particle size and higher yield can be obtained at a convergence angle of 32°, an extension length of 5 mm, a melt temperature of 800 °C, and an atomization pressure of 3.25 MPa. For the powder prepared under the optimum processing parameters, testing including chemical composition, morphology, apparent and tap density, and Hall flow rate was conducted for further performance analysis. The chemical composition is shown in Table 9. According to the designed chemical composition and the test result, there is a significant difference in the content of Mg element, which is mainly due to the burning during the melting process.

**Figure 10:**
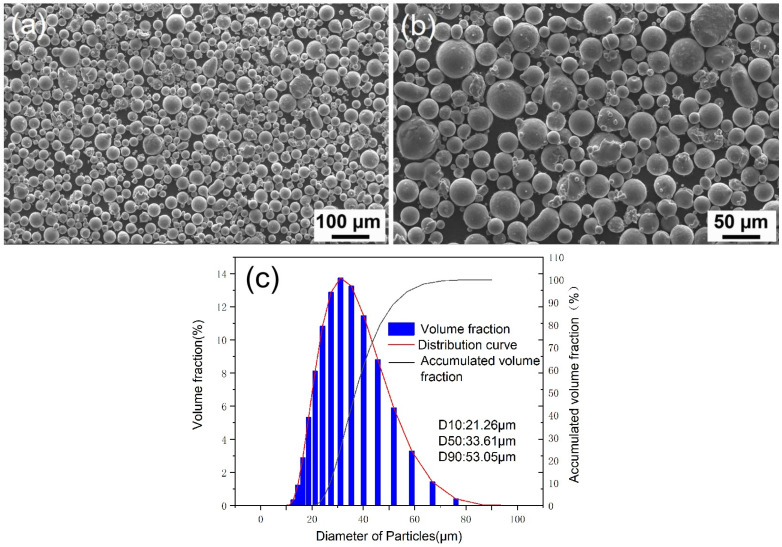
Morphology of the prepared Al-Mg-Er-Zr-Sc alloy powder (**a**) and the enlarged figure (**b**); (**c**) the particle size distribution.

Figure 10 shows the powder morphology and the particle size distribution. It can be seen that the particles are spherical or nearly spherical, with a few small particles adhering to the surface of some large particles. In the atomization process of VIGA, high-pressure gas breaks the metal melt into small droplets. During the high-speed flight, on the one hand, the metal droplets exchange heat with the surrounding environment and gradually solidify as the temperature decreases. On the other hand, under the action of surface tension, metal droplets tend to spheroidize in order to reduce the surface energy. Due to the fact that the spheroidization time of melt droplets is much shorter than the solidification time, the spheroidization evolution is completed before it fully solidifies. Additionally, there exists a small number of elongated particles, which is related to the cooling and solidification conditions during the atomization process [32].

**Figure 11:**
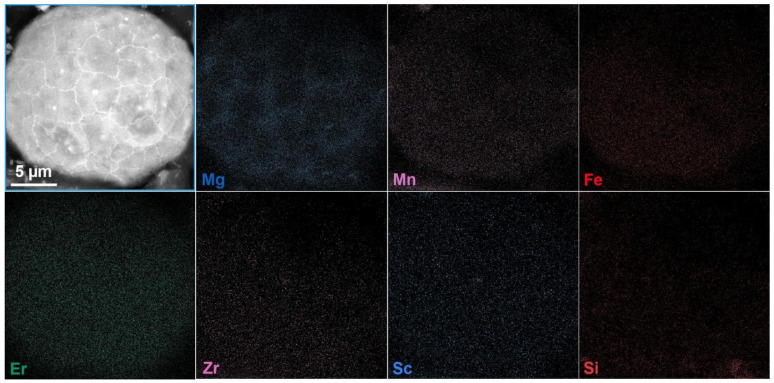
The microstructure and SEM-EDX mapping images of the prepared Al-Mg-Er-Zr-Sc alloy powder.

The microstructure and SEM-EDX mapping images of the powder particle are shown in Figure 11. The powder particle is composed of many small grains, with an average grain size of about 3.5 μm. And Al_3_M (M is one or several elements of Er, Sc, and Zr) phases can be observed in some of the grains, which precipitate in the cooling process of droplets during atomization. Except for the slight segregation of Mg along grain boundaries, other elements are uniformly distributed in the powder particle. When the Mg content exceeds 3.5%, the Mg element tends to precipitate in the form of the Al_3_Mg_2_ phase at the grain boundaries [33]. The Al_3_Mg_2_ has a high nucleation energy barrier, and the grain boundaries provide favorable nucleation sites due to the high free energy. Table 10 shows the physical properties of the powder. The apparent density and tap density are 1.13 g/cm^3^ and 1.36 g/cm^3^, respectively, and the Hall flow rate is 75 s/50 g.

### 4.4. LPBF-Processed Part Performance

**Figure 12:**
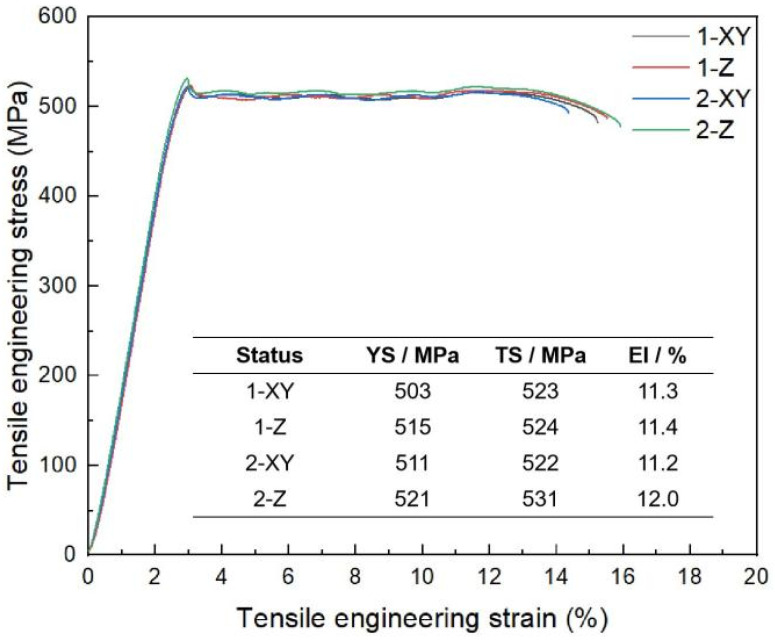
Mechanical properties of the LPBF-processed Al-Mg-Er-Zr-Sc aluminum alloy.

Figure 12 presents the mechanical properties of the LPBF-processed Al-Mg-Er-Zr-Sc aluminum alloy, with XY representing the horizontal samples and Z representing the vertical samples. Both the horizontal and vertical samples were subjected to a heat treatment (350 °C/2 h) before the room temperature tensile test. The results show that the tensile strength of samples is above 520 MPa, the yield strength is above 500 MPa, and the elongation is above 11%. The mechanical properties of the studied alloy are comparable to the Scalmalloy alloy developed by Airbus, and the yield strength is much higher by comparison. In addition, it is worth noting that there is a stress drop after the yield on the stress–strain curves, which has also been found in AlMgScZr alloys [2] and is associated with the interactions between precipitates and dislocations. Dislocations form during the LPBF process because of the large thermal stress that exists in the as-built samples. During the heat treatment, nano-Al_3_M particles tend to precipitate along the dislocations from the supersaturated Al matrix. The dislocations are hindered in their motion and a slightly higher stress is required to tear them off. Once they are released, a rapid stress drop takes place as shown on the stress–strain curves.

## 5. Conclusions

In this work, a new type atomizer with an optimized convergence angle and tube extension length suitable for Al-Mg-Er-Zr-Sc aluminum alloy was designed by a combination of numerical simulation and experimental methods, and the optimum atomization processing parameters were determined.

1. When the convergence angle was 32° and the extension length was 5 mm, the large negative pressure and suction force at the tube outlet could facilitate the smooth flow of the melt and a suitable powder particle size distribution was obtained.
2. When the melt temperature was 800 °C and the atomization pressure was 3.25 Mpa, the melt had low viscosity and the atomization gas could fully interact with the melt. Meanwhile, atomized droplets had suitable cooling conditions, avoiding the generation of irregular powder and improving the powder sphericity. Under the optimal processing parameters, the prepared powders were spherical or nearly spherical with fine particle size and a high yield of about 39.45%.
3. The LPBF-processed Al-Mg-Er-Zr-Sc high-strength aluminum alloy exhibited excellent mechanical properties, with a tensile strength of over 520 MPa, a yield strength of over 500 MPa, and an elongation of over 11%.

## Figures and Tables

**Table 1:** High-strength aluminum alloy materials currently used in additive manufacturing [2,3,4,5,6].

Number	Material System	Brand	Company	Tensile Strength /MPa	Yield Strength /MPa	Elongation /%
1	Al-Mg-Er-Zr	HSAL-13	Avimetal-am	520	500	11–13%
2	Al-Cu-TiB2	AM205	Aeromet International	450–511	390–440	10–13%
3	Al-Mg-Sc-Zr	Scalmalloy	The Airbus Apworks	520	480	13%
4	Al-Mg-Sc-Zr	HAlSc	Changsha New Material Industry Research Institute	535	510	12%
5	Al-Mg-Sc-Zr	CRRC-HAP-1	CRRC Academy	560	500	10%
6	Al-Mg-Sc-Zr	HS5501	Baohang materials	530	520	10%
7	Al-Mn-Sc-Zr	Al250C	SuZhou Am proinnovations	590	580	11%
8	Al-Cu-Ag	AM 2139	EOS	520–540	460	4–6%

**Table 2:** Designed chemical composition of high-strength aluminum alloy.

Element	Al	Mg	Er	Zr	Sc	Fe	Si	Mn
Mass ratio (%)	Bal.	4.50	0.70	0.50	0.30	0.30	0.30	0.50

**Table 3:** LPBF processing parameters.

Parameter	Laser Power	Scan Speed	Scan Spacing	Layer Thickness	Fill Angle
Numerical value	280 W	1200 mm/s	0.08 mm	30 μm	67°

**Table 4:** Orthogonal parameters with different convergence angles.

Number	Convergence Angle/°	Extension Length/mm
2–1	28	6
2–2	32	6
2–3	36	6
2–4	40	6
2–5	44	6
2–6	48	6

**Table 5:** Orthogonal parameters with different extension lengths.

Number	Convergence Angle/°	Extension Length/mm
2–1	28	5
2–2	28	6
2–3	28	7
2–4	30	5
2–5	30	6
2–6	30	7
2–7	32	5
2–8	32	6
2–9	32	7

**Table 6:** Median particle size and powder yield under different convergence angles and extension lengths.

Number	Convergence Angle/°	Extension Length/mm	D50/μm	n*_ρ_*/%
2-1	28	5	46.43	32.64.
2-2	28	6	49.25	31.58
2-3	28	7	54.02	29.31
2-4	30	5	45.24	33.59
2-5	30	6	47.45	32.15
2-6	30	7	52.53	30.02
2-7	32	5	44.65	34.53
2-8	32	6	48.27	31.79
2-9	32	7	56.25	28.56

**Table 7:** Orthogonal parameters with different melt temperatures and atomization pressures.

Number	Melt Temperature/°C	Atomization Pressure/MPa
3-1	750	3.0
3-2	750	3.25
3-3	750	3.50
3-4	800	3.0
3-5	800	3.25
3-6	800	3.50
3-7	850	3.0
3-8	850	3.25
3-9	850	3.50

**Table 8:** Median particle size and powder yield under different melt temperatures and atomization pressures.

Number	Melt Temperature/°C	Atomization Pressure/MPa	D50/μm	n*_ρ_*/%
3-1	750	3.0	44.65	34.53
3-2	750	3.25	41.98	36.28
3-3	750	3.50	40.13	36.98
3-4	800	3.0	39.26	37.54
3-5	800	3.25	37.57	39.45
3-6	800	3.50	38.64	38.03
3-7	850	3.0	43.44	35.26
3-8	850	3.25	42.03	36.03
3-9	850	3.50	41.08	36.55

**Table 9:** Chemical composition of the prepared Al-Mg-Er-Zr-Sc alloy powder.

Element	Al	Mg	Er	Zr	Sc	Fe	Si	Mn	O	N
Mass ratio (%)	bal	4.15	0.66	0.47	0.31	0.28	0.19	0.53	0.035	0.012

**Table 10:** The physical properties of the prepared Al-Mg-Er-Zr-Sc alloy powder.

Test Indicators	Apparent Density/g/cm^3^	Tap Density/g/cm^3^	Hall Flow Rate/s/50 g
Numerical value	1.13	1.36	75

## Data Availability

The original contributions presented in this study are included in the article. Further inquiries can be directed to the corresponding authors.
